# A Case of Milk-Alkali Syndrome

**DOI:** 10.7759/cureus.38171

**Published:** 2023-04-26

**Authors:** Isaac Opoku, Njika Atemnkeng, Nedsely Vila

**Affiliations:** 1 Internal Medicine, Piedmont Athens Regional, Athens, USA

**Keywords:** hypercalcemia, calcium, syndrome, alkali, milk

## Abstract

Milk-alkali syndrome is described by a triad of elevated levels of calcium, metabolic alkalosis, and acute kidney injury that historically occurred as a result of the combined intake of large amounts of calcium and absorbable alkali. It is becoming common recently with the use of over-the-counter calcium supplements for osteoporosis treatment in postmenopausal women. We present a case of a 62-year-old female who presented with generalized weakness. She was noted to have severe hypercalcemia, and impaired renal function with a significant history of daily over-the-counter calcium supplement use and as-needed calcium carbonate use for gastroesophageal reflux disease (GERD). This case highlights the stepwise approach to the evaluation and management of hypercalcemia. She was appropriately treated with the resolution of hypercalcemia and presenting symptoms.

## Introduction

Characterized by the triad of elevated levels of calcium, metabolic alkalosis, and acute kidney injury, milk-alkali syndrome commonly occurs due to the combined intake of large amounts of calcium and absorbable alkali [1]. Historically, this syndrome was commonly seen when Dr. Bertram Sippy introduced peptic ulcer disease treatment consisting of milk and cream combined with absorbable alkali [1,2]. With the advent of modern histamine (H2)-antagonists and proton pump inhibitor (PPI) treatment for peptic ulcer disease, this syndrome virtually vanished [1]. Recently, however, an increased number of cases of milk-alkali syndrome have been reported. This is likely due to the common use of over-the-counter calcium preparations for preventing and treating osteoporosis in postmenopausal women [1]. Milk-alkali syndrome is becoming a more frequent cause of hypercalcemia. This case report is to create awareness of this syndrome among physicians and to highlight the stepwise approach to its evaluation and treatment.

## Case presentation

The patient was a 62-year-old female who was brought into the emergency department with a five-day history of generalized weakness and poor oral intake. She had no other symptoms. Her past medical history includes hypertension, gastroesophageal reflux disease (GERD), and osteoarthritis of the knees and hips. She was on an over-the-counter calcium-vitamin D3 supplement for osteoporosis. Each tablet of this supplement consists of 600mg of elemental calcium and 20mcg of vitamin D3. She was taking one tablet twice a day, making a total of 1200mg per day for over two years. The patient was also on as-needed calcium carbonate antacid, which she was taking as two tablets three times a day for six weeks prior to presentation. One tablet of this antacid contains 400mg of calcium, making a total of 2400mg of elemental calcium per day. In total, the patient was taking 3600mg of elemental calcium per day. She was also on as-needed tramadol for osteoarthritis, and hypertension which was managed non-pharmacologically.

At presentation, the patient was conscious and oriented in time, place, and person. Her blood pressure was mildly elevated at 147/102. She had tachycardia of 108. Other vitals were stable. Physical examination revealed tenderness in the right hip and lower back regions but no swelling or bruising was noted. Other systems were normal.

Blood work showed hypercalcemia with a total calcium level >18mg /dL, phosphorus low at 2.0mg/dl, hypokalemia of 2.5mmol/L, bicarbonate of 28, and creatinine of 1.23mg/dl. Parathyroid hormone (PTH) was low at 8.70pg/ml. Other workups for hypercalcemia included parathyroid hormone-related protein (PTHrP); 1,25-hydroxy (OH) vitamin D; 25-OH vitamin D; serum protein electrophoresis (SPEP); and free light chains (kappa and lambda) were unremarkable. A complete blood count (CBC) showed leukocytosis.

**Figure 1:**
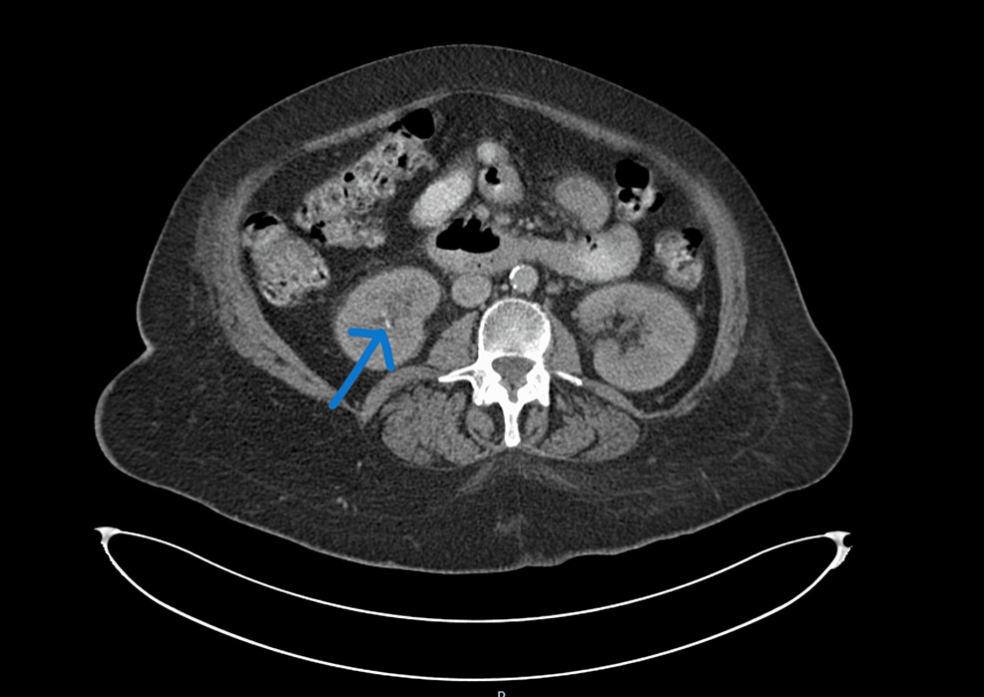
Axial view of the 4mm non-obstructing stone in the right kidney on the CT scan

**Figure 2:**
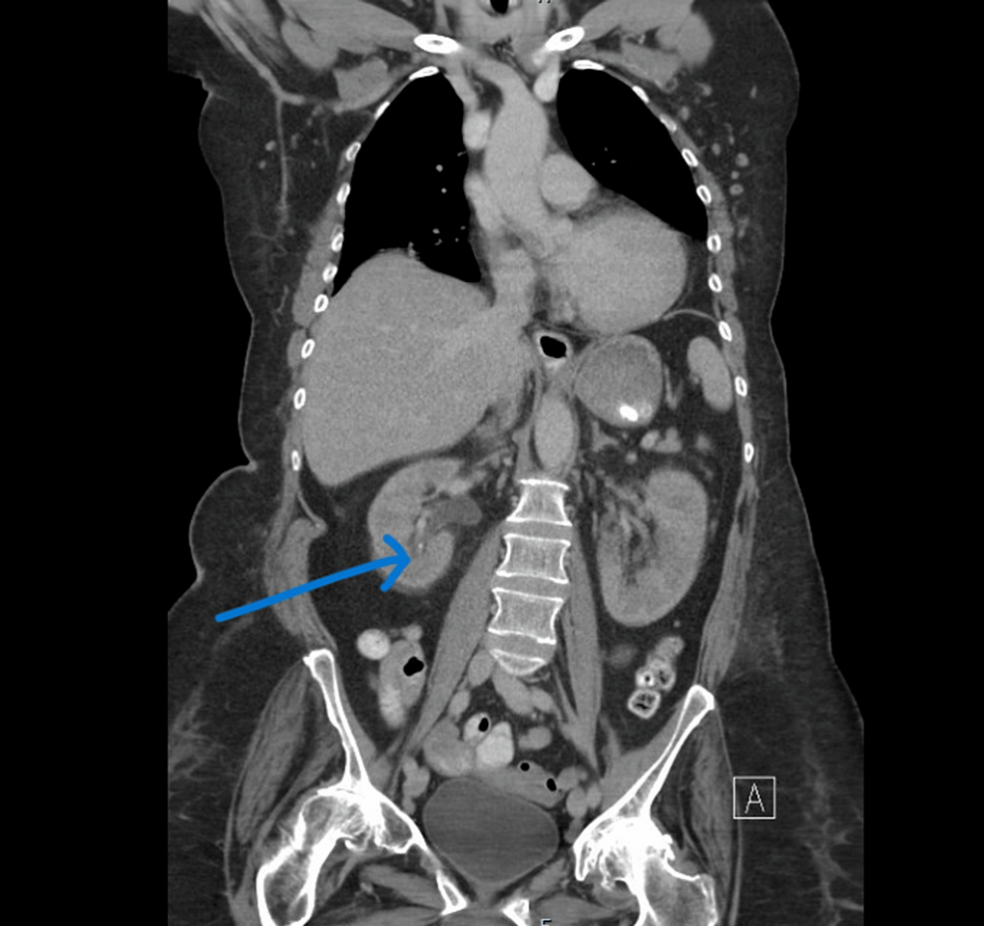
Coronal view of the 4mm non-obstructing stone in the inferior collecting system of the right kidney on the CT scan

The CT chest, abdomen, and pelvis with IV contrast to evaluate for malignancy was unremarkable except for a 4mm non-obstructing stone in the collecting system of the right kidney (Figures 1-2). Non-contrast CT head and electrocardiogram (EKG) were unremarkable. The X-rays of the lumbar-sacral spine, pelvis, hips, and knees showed no fracture or dislocation but revealed chronic degenerative bone disease suggestive of lumbar spondylosis and hip osteoarthritis. 

Table 1 shows the results of monitoring renal function, electrolytes, hemoglobin, white cell count, and platelet count. Table 2 and Table 3 show the results of hypercalcemia workup and SPEP results, respectively. 

**Table 1:** Results of serial measurements of calcium, creatinine, complete blood count, and other electrolytes Ca: Calcium

Laboratory test	Reference range	Day 1	Day 2	Day 3	Day 4	Day 5
Total Ca	8.4-10.2mg/dL	>18	15.3	13.8	11.3	9.7
Potassium	3.3-5.1mmol/L	2.5	3.9	4.0	3.8	
Creatinine	0.40-1.00mg/dL	1.23	1.40	1.43	1.28	1.24
Bicarbonate	22-32mmol/L	28	27	24	25	
Phosphorus	2.6-4.5mg/dL	2.00		2.1	2.6	
Hemoglobin	12.0-16.0g/dL	12.0	10.7	9.7	9.7	9.7
White cell count	4.0-10.5 x 10^3^/uL	20.5	15.5	13.6	11.0	10.1
Platelet	130-400 x10^3^/uL	447	363	328	332	352

**Table 2:** Laboratory results for hypercalcemia workup PTH: Parathyroid hormone, PTHrP: Parathyroid hormone-related peptide, 25-OH Vitamin D: 25-hydroxy-vitamin D

Lab test	Results	Reference range
PTH	8.70pg/mL	15.00-80.00pg/mL
PTHrP	10pg/mL	11-20pg/mL
25-OH Vitamin D	33.2ng/mL	30.0-100.0ng/mL
Free kappa	18.0	3.3-19.4mg/L
Free lambda	19.9	5.7-26.3mg/L
Free kappa/lambda Ratio	0.90	0.26-1.65

**Table 3:** Serum protein electrophoresis

Component	Results	Reference range
Total protein	6.5	6.1-8.1g/dL
Alpha 1	0.4	0.2-0.3g/dL
Alpha 2	0.9	0.5-0.9g/dL
Beta-1	0.5	0.4-0.6g/dL
Beta-2	0.5	0.2-0.5g/dL
Gamma globulin	0.8	0.8-1.7g/dL
Albumin	3.6	3.8-4.8g/dL
Abnormal protein band	Not detected	

The patient received aggressive 0.9% sodium chloride (NaCl) intravenous fluid resuscitation and monitoring of urine output for hypercalcemia, with serial calcium checks. In addition, she received IV calcitonin and zoledronic acid. Her home calcium carbonate and calcium supplements were stopped on presentation. Other electrolyte abnormalities were corrected (hypophosphatemia and hypokalemia). Her renal function was monitored closely as shown in Table 1, and nephrotoxins were avoided. In all, the presenting symptoms and hypercalcemia resolved completely. She was discharged to subacute rehab. The serum calcium check one week after discharge was 9.0mg/dL.

## Discussion

Milk-alkali syndrome was first described in the 20th century when Dr. Bertram Sippy introduced the ‘Sippy regimen’ for peptic ulcer treatment [3]. This regimen consisted of multiple daily doses of milk combined with absorbable alkalis such as magnesium oxide, sodium bicarbonate, or bismuth subcarbonate. Results were initially good and became a popular therapy. However, there were reports of people on this therapy developing acute kidney injury, and metabolic alkalosis [2]. Cope then described hypercalcemia as an element of the milk-alkali syndrome [4]. With the introduction of new drugs for peptic ulcer treatment such as H2 receptor blockers, the syndrome vanished. There has, however, been an increased number of cases in recent times due to the common use of over-the-counter preparations of calcium for the prevention of osteoporosis in postmenopausal women [1,5]. Calcium carbonate is also frequently prescribed in patients with chronic kidney disease, for peptic ulcer disease, or the prevention of secondary hyperparathyroidism [1]. Similarly, our patient is postmenopausal and was on calcium carbonate for acid reflux and an over-the-counter calcium supplement before developing this syndrome.

Some scholars have suggested changing the name to calcium-alkali syndrome due to the etiopathology of the current syndrome which differs from the classical milk-alkali syndrome described with Sippy’s regimen [1]. The modern form of milk-alkali syndrome is female-dominated with an average age of 50 years compared to the classical form which was common in middle-aged males [5]. The modern form is mainly caused by over-the-counter calcium carbonate [1,5]. A similar case was reported about a 47-year-old woman who presented with renal stones and hypercalcemia from supplemental calcium [6]. Milk-alkali syndrome now accounts for more than 10% of the cases of hypercalcemia and is the third most common cause of hypercalcemia in hospitalized patients, after hyperparathyroidism and malignancy [1,5].

Diagnosis of milk-alkali syndrome requires a history of excessive calcium and alkali use, findings of hypercalcemia on blood work, and kidney injury that are not due to other causes [7]. There are three forms of milk-alkali syndrome described, namely acute, subacute, and chronic but they do overlap. Some early symptoms include nausea, vomiting, headache, dizziness, irritability, apathy, and confusion. For the chronic phase, symptoms include muscle aches, psychosis, tremor, polyuria, polydipsia, or pruritus. Renal calcinosis is not uncommon [7].

Hypercalcemia is always present. Alkalotic pH, elevated bicarbonate, and kidney injury are usually present [7]. Hypercalcemia has various effects on the kidney, eventually resulting in hypovolemia. It decreases the glomerular filtration rate (GFR) by causing vasoconstriction and natriuresis by indirectly inhibiting the sodium-potassium-chloride(Na-K-2Cl) channels in the medullary thick ascending limb of the nephron. It also decreases water reabsorption by inhibiting the antidiuretic hormone receptors (V2 receptors) in the basolateral membrane of collecting tubules in the kidney. The resultant hypovolemia contributes to the development of alkalosis by increasing bicarbonate reabsorption. Alkalosis itself leads to increased calcium reabsorption from the nephron's distal tubule, leading to hypercalcemia. A vicious cycle, therefore, develops due to hypercalcemia, alkalosis, and kidney injury [1]. Our patient had hypercalcemia and kidney injury. Her bicarbonate at presentation was close to the upper limit of the normal range. A low to normal phosphate level is usually seen in the modern form of the syndrome as seen in our patient who presented with a phosphate level of 2.0mg/dL (reference range: 2.6-4.5mg/dL). The classic form has hyperphosphatemia [7]. Intact PTH levels are decreased just as in our patient with a value of 8.70pg/mL (reference range: 15.00-80.00pg/mL).

Withdrawal of the offending agent, hydration, and supportive therapy are generally sufficient treatments. The patient may recover in one to two days in the acute form. Recovery in chronic forms is a slower process. Hemodialysis may be needed in refractory cases. Furosemide may be used to enhance calciuresis [7]. Daily elemental calcium intake of no more than 2g is considered safe [8]. Reducing daily calcium intake may be necessary in patients taking thiazides [9], and in patients who have pre-existing renal failure [10]. Our patient received IV fluids, calcitonin, and zoledronic acid. Calcium carbonate and calcium supplements which were considered the offending medications were stopped. Our patient’s calcium and phosphorus returned to normal levels in four to five days. Her renal function had also improved at the time of discharge. 

## Conclusions

The current form of milk-alkali syndrome is due to the increased use of calcium supplements as a result of osteoporosis awareness. The exact mechanism of this syndrome is unknown and it can lead to permanent kidney injury and other complications of hypercalcemia. Treatment is supportive by the removal of offending agents and hydration. Both physicians and the public need to be aware of the potential adverse effects of ingesting excessive amounts of calcium.
